# Assessment of *Plasmodium vivax* transmission and asymptomatic carriage risk among artisanal gold miners in western French Guiana, 2014–2020

**DOI:** 10.1186/s40249-025-01306-8

**Published:** 2025-05-26

**Authors:** Alice Sanna, Yann Lambert, Stéphane Pelleau, Lise Musset, Yassamine Lazrek, Louise Hureau, Hedley Cairo, Stephen Vreden, Michael White, Maylis Douine

**Affiliations:** 1 Centre d’Investigation Clinique (Inserm 1424), Institut Santé des Populations en Amazonie, Centre Hospitalier de Cayenne, Cayenne, French Guiana; 2 Infectious Disease Epidemiology and Analytics, Institut Pasteur, Université Paris Cité, Paris, France; 3 Parasitology Laboratory, National Malaria Reference Center, Institut Pasteur de La Guyane, Cayenne, French Guiana; 4 National Malaria Programme, Ministry of Health, Paramaribo, Suriname; 5 Foundation for the Advancement of Scientific Research in Suriname (SWOS), Paramaribo, Suriname

**Keywords:** Malaria elimination, *Plasmodium vivax*, Prevalence, Incidence, Serology, Asymptomatic carriage, Hard-to-reach population

## Abstract

**Background:**

The final challenge for malaria elimination in many countries is to interrupt the circulation of *Plasmodium vivax*. Given the unique biology of this parasite, innovative approaches are imperative, with a focus on identifying asymptomatic carriers of dormant parasite forms. This article delineates the recent epidemiological patterns of *P. vivax* malaria within a highly mobile and hard-to-reach population in the Guiana Shield. It further proposes an assessment of the potential reservoir of asymptomatic carriers.

**Methods:**

This analysis was based on data from: (i) two cross-sectional surveys carried out at the French-Surinamese border in 2015 and 2019, including adults returning from gold mining sites located in French Guiana (FG), [questionnaires and blood samples, tested for polymerase chain reaction (PCR) and *P. vivax* serological exposure markers (SEM) of recent infection]; (ii) epidemiological malaria surveillance system in Suriname, including cases imported from gold mining sites located in western FG between 2014 and 2020. Factors associated with *P. vivax* seropositivity were analysed by multiple logistic regression. The probability of carrying *P. vivax* parasites (blood-stage or hypnozoite) was estimated by a classification drawn from PCR results, SEM and reported recent history of illness.

**Results:**

Surveillance data showed a decrease in malaria imported cases from French Guiana between beginning and end of the analysed period (236 in 2014 to 74 in 2020) and an increase in the proportion of cases associated with *P. vivax* (52.4% in 2014 to 100% in 2020). The PCR-prevalence of *P. vivax* in survey samples decreased from 11.4% in 2015 to 4.0% in 2019; *P. vivax* seropositivity decreased from 44.7% to 28.4%. *P. vivax* seropositivity was positively associated with male sex, age and number of years spent in gold mining, type of activity, and reported malaria history (episode within less than nine months *OR* = 10.73, 95% *CI*: 5.87–19.6, or history of repeated older episodes *OR* = 5.31, 95% *CI*: 3.13–9.01).

**Conclusions:**

Our analysis shows an epidemiological evolution typical of a scenario of decreasing malaria circulation. Nevertheless, in 2020, gold miners in western FG still showed a moderate level of *P. vivax* circulation. Biological methods and epidemiological criteria can help to select potential parasite carriers, who could benefit from targeted drug administration.

**Graphical Abstract:**

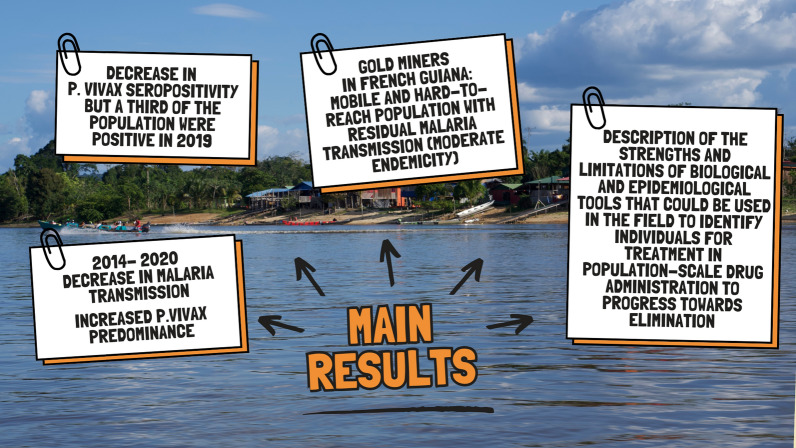

**Supplementary Information:**

The online version contains supplementary material available at 10.1186/s40249-025-01306-8.

## Background

Malaria elimination is a major public health goal that is becoming an achievable reality in several countries around the world [1, 2]. In many of these countries, residual malaria transmission in hard-to-reach populations is a major challenge, that is further complicated by the circulation of *Plasmodium vivax* [3–5].

*P. vivax* has several biological characteristics that facilitate its persistence and resilience to conventional case management strategies, including the hepatic persistence of hypnozoites, that trigger periodically recurrent malaria attacks or asymptomatic parasitaemia. To date, there is no diagnostic method to identify hypnozoite carriage. Recurrences associated with hypnozoite reactivation can be prevented by treating patients with 8-aminoquinolines in addition to acute treatment with drugs targeting circulating forms [6–8].

Malaria serology is an old and multi-faceted method, whose role in supporting elimination has recently been revisited, particularly in light of the challenges posed by *P. vivax* [9]. Serology can be used to study the exposure of a population to parasites, and thus provide useful epidemiological indicators for risk stratification and to determine the current state of transmission [10]. In the case of *P. vivax,* it is also currently proposed as a tool to detect the likelihood of recent infections using a panel of selected antibodies [serological exposure markers (SEMs)], and can therefore represent a proxy for assessing the risk of hypnozoites carriage [11]. The performance of this method in identifying recently exposed individuals varies according to the level of endemicity of the target population, with its best performance in low transmission settings: in medium and high transmission contexts, the SEM is less accurate in distinguishing between individuals with a recent infection (≤ 9 months) and those with an immunological memory associated with a long-term exposure [12].

Early diagnosis and treatment, integrated vector control, surveillance and case management must be the core strategy for malaria elimination everywhere, according to the World Health Organization (WHO). In areas of low or moderate incidence, it is also recommended to implement population-based interventions that target the human reservoir of parasites and that have the potential to interrupt transmission [13]. Among these interventions, mass drug administration (MDA) may have a questionable risk–benefit ratio, especially in cases of low transmission and/or when targeting *P. vivax*. In this case, 8-aminoquinolines in addition to schizonticidal treatment should be used (conditional favourable recommendation by WHO) [14, 15]. In these contexts, SEMs could be used to better target the members of a community who should be offered treatment [*P. vivax* serological test and treatment (SeroTaT)] [11, 16]. Alternatively, selection can be based on epidemiological criteria of belonging to a high risk group for targeted drug administration (TDA) [13, 17].

French Guiana (France) and Suriname are two contiguous territories, located in South America and largely covered by the Amazon rainforest. Malaria (*P. falciparum*, *P. vivax* and *P. malariae*) has been endemic in the region for several centuries, but thanks to local and joint efforts, the incidence of new cases has decreased significantly over the last decade: from 569 indigenous cases reported in 2012 to zero in 2022 in Suriname, and from 900 to 21 in French Guiana [1]. They are among the areas selected by the WHO as candidates for malaria elimination by 2025 (E-2025 initiative) [1]. Residual transmission of malaria occurs mainly among people involved in artisanal and small-scale gold mining (ASGM) in the heart of the tropical forest, particularly those working in French Guiana. This is because their activity is very often clandestine and is repressed by the French authorities, which creates several obstacles (operational, regulatory and political) to the provision of care and prevention on the mining sites [18–20]. Most of these people are of Brazilian origin, and the French-Surinamese border is often their gateway to the sites located in French Guiana. This community also commutes regularly between the sites in French Guiana and the supply centres are located along the border or in the Surinamese capital of Paramaribo [21–23]. When they leave the forest, gold miners tend to seek malaria care at Surinamese facilities rather than French health centres located on the border. Despite improvements in regional epidemiology during the last decade, this specific population has shown a high to moderate level of malaria transmission [20, 24]. Several initiatives are attempting to provide solutions to malaria circulation in this challenging cross-border context, including two intervention research projects: the Malakit project (2018–2020) and the CUREMA project [*Radical CURE for MAlaria among highly mobile and hard-to-reach populations in the Guiana Shield],* 2022–ongoing, described in detail elsewhere [24–26]. The CUREMA project aims to evaluate a complex intervention that includes offering a TDA to gold miners considered to be at risk of asymptomatic *P. vivax* carriage, to reduce the risk of relapse and transmission of the parasite. In the absence of a serological test accessible in the remote regions where the project is implemented, the selection of individuals to be treated is based on a combination of epidemiological criteria [26].

The aim of this article is to describe recent trends in *P. vivax* malaria transmission in the ASGM community of western French Guiana. It also aims to estimate the risk of asymptomatic carriage of *P. vivax* using different approaches such as molecular biology, serology and epidemiological criteria. This will provide an insight into the relevance of TDA approaches, such as those used in the case of the CUREMA project.

## Methods

### Data sources

The data presented in this analysis is derived from two sources: epidemiological surveys and surveillance systems.

Two cross-sectional surveys, ORPAL 1 ([ORpaillage et PALudisme] *Prevalence survey of malaria parasites in people working in illegal gold mining in French Guiana***)** and ORPAL 2] were conducted from January to May 2015 and from September to December 2019, respectively. These surveys were conducted on the French-Surinamese border, in settlements that housed supply centers of the ASGM community. Individuals 18 years of age and over who had returned from a gold mining site located in French Guiana within the last seven days were included in the surveys. The inclusion process was performed by a doctor, a registered nurse and a facilitator (a Portuguese-speaking person belonging or closely associated with the community). A questionnaire was used to collect socio-demographic and mobility information, knowledge and behaviours regarding malaria, recent malaria history and comorbidities. Venous blood sampling and a medical examination were systematically conducted. More information on these surveys has been previously published [20, 24, 25].

Suriname’s national malaria surveillance system collected data about malaria cases confirmed by microscopy or rapid diagnostic test (RDT), resulting from passive surveillance and (re)active case detection, as well as epidemiological investigation regarding the place of infection of each case. For the purposes of this particular analysis, cases that had been notified between 2014 and 2020 and had been classified as imported from French Guiana were selected.

**Fig. 1:**
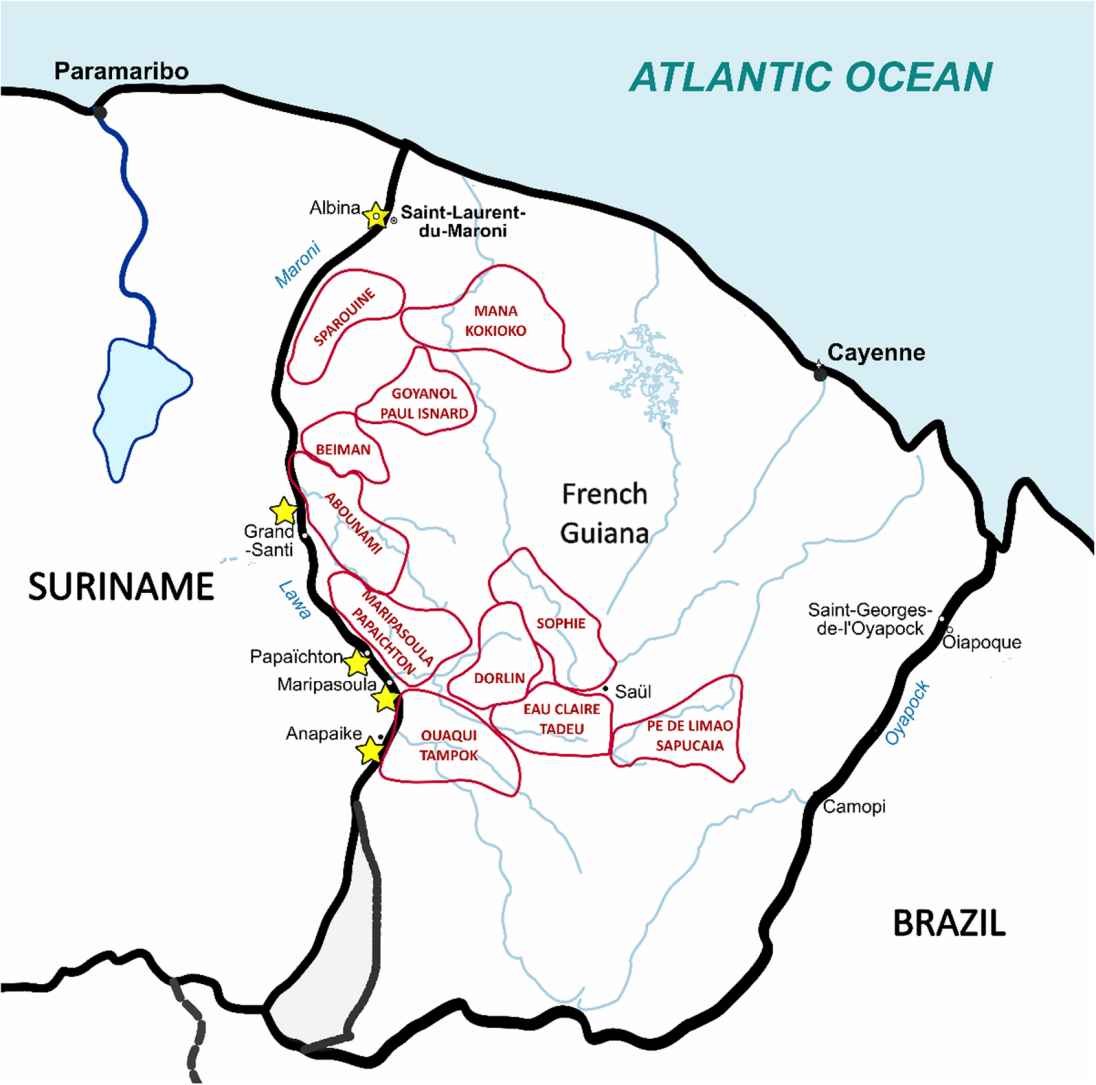
Map of French Guiana illustrating the main gold mining areas that have been selected for the present analysis (red shapes) and inclusion sites for cross-sectional surveys (yellow stars) (modified from https://d-maps.com/)

For both sources, a selection was made of cases linked to the gold mining areas of western French Guiana (Fig. 1). Cases from the epidemiological surveys were people who had returned from these areas within the previous seven days; cases notified in the Surinamese surveillance system were linked to these sites following an epidemiological investigation.

### Laboratory analyses from cross-sectional surveys

Venous blood samples were collected from each participant in the cross-sectional surveys and stored at 28 °C for a maximum of five days. Subsequently, these samples were transferred to the parasitology laboratory of the Institut Pasteur de la Guyane (IPG) for malaria diagnosis using a standard nested-PCR (polymerase chain reaction) targeting the 18S ribosomal deoxyribonucleic acid [rDNA] on DNA extracted with the QIAamp^®^ DNA kit (Qiagen) from 200 µl of blood [27].

Sera aliquots were then frozen at − 80 °C and subsequently transferred to the Institut Pasteur in Paris for the serological analyses, which were realised using a multiplex Luminex Magpix platform to measure antibodies against *P. vivax* proteins. The panel contained eight *P. vivax* blood-stage antigens [PVX_094255B (RBP2b), PVX_094255 A (RBP2b), PVX_087885B (RAMA, putative), PVX_099980 (MSP1-19), PVX_112670 (unspecified), PVX_096995 (Pv-fam-a), KMZ83376.1 (PvEBPII), PVX_097625 (MSP8, putative), PVX_097720 (MSP3.10)], as identified by Longely et al. [11], with the antigen most strongly associated with recent blood-stage infection being *P. vivax* reticulocyte binding protein 2b (RBP2b). Measured IgG antibody levels were analysed using a previously described algorithm [11] to provide a classification of sero-negative or sero-positive. Firstly, seropositivity was defined using the thresholds identified during model calibration to obtain a sensitivity of 63% and a specificity of 90% (SEM: 63–90) for the detection of recent (9 months or less) *P. vivax* exposure, which was deemed suitable for describing the seroprevalence associated with recent infection in a population characterised by medium-intensity transmission and a history of significant exposure to malaria [12]. Secondly, a seropositivity indicator was also produced using thresholds identified to obtain a sensitivity and specificity of 79% (SEM: 79–79). These parameters were considered more performant for identifying people to be treated during pharmacological interventions such as *P. vivax* SeroTAT [11, 16]. The distribution of log-values for antibodies directed against the *P. vivax* RBP2b.F2 antigen was also described.

The present study exclusively included subjects for whom both PCR and serological test results were available.

### Statistical analyses

The data were analysed using R software (v 4.4.0, The R Foundation for Statistical Computing, Vienna, Austria) with RStudio integrated development environment (Posit Software, Boston, USA). Quantitative data were described both in terms of mean, median, standard deviation, and minimum and maximum values; categorical variables were described in terms of numbers and proportions. Univariable analyses were performed for these two types of variables using the Wilcoxon and Fisher tests respectively.

Exploratory analysis of factors associated with seropositivity (SEM: 79–79) was performed using a multiple logistic regression model. The initial model incorporated a purposive selection of variables following a comprehensive evaluation of the univariable analysis outcomes, potential confounding factors, and the linearity of the relationship between the variable of interest and its explanatory variables when pertinent. The final model was constructed using a stepwise descent method.

Given the potential limitations of SEM methods in the endemicity level of the study population, a composite score was created to explore the likelihood of recent *P. vivax* infection using the data available from the two cross-sectional surveys. The score takes into account biological and epidemiological criteria, and individuals are classified according to a criterion of decreasing probability of carrying *P. vivax* (in blood-stage or hypnozoite form). The values of this score are defined as follows:

- *Confirmed*: PCR positive individuals (either symptomatic or asymptomatic)
- *Probable*: PCR negative individuals
  - Either reporting a malaria episode with positive testing in the last 9 months;
  - Or reporting a malaria-like symptomatic episode without a biological diagnosis in the last 9 months but with positive SEM (79–79).
- *Possible*: PCR negative individuals
  - Either not reporting any malaria symptoms episode in the last 9 months but SEM (79–79) positive;
  - Or reporting a malaria symptoms episode in the last 9 months but SEM (79–79) negative.
- *Unlikely*: Individuals who are PCR negative, SEM (79–79) negative and have not reported any history of malaria symptoms in the last 9 months.

The composite score thus constructed enabled the establishment of three categories indicating whether individuals who should be offered treatment in the event of a pharmaceutical population-scale intervention targeting the human reservoir of *P. vivax*: those with a confirmed or probable recent infection should be treated; those with an unlikely recent infection should not be treated; and those with doubt about the relevance of offering treatment to people with a possible recent infection.

These categories were then compared with approaches potentially used to select individuals for treatment: PCR, serology (SEM: 79–79), TDA treating all the persons declaring a recent (≤ 9 months) malaria episode, TDA with the risk assessment criterion used in the CUREMA intervention, TDA treating the overall high-risk population (individuals involved in ASGM in French Guiana). The objective of this comparison is to estimate the proportion of carriers missed and the risk of overtreatment for each approach, in our study sample. The CUREMA intervention criterium was defined as follows: report at least one malaria symptoms episode in the previous 12 months or have a lifelong malaria history and have been in a high-endemicity area in the previous 12 months (in this case, any mining area in the region except for Suriname mines).

## Results

### Population characteristics

**Fig. 2:**
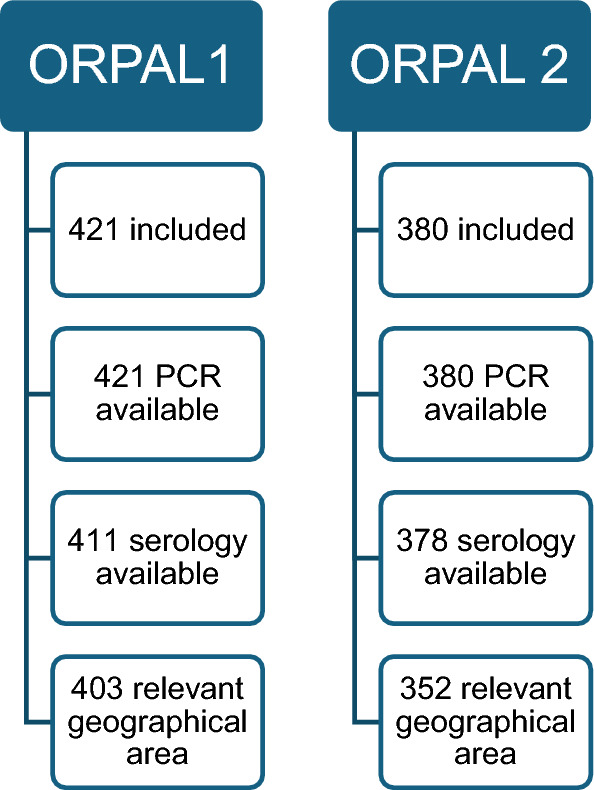
Participants selection in the cross-sectional surveys: among the participants included in the surveys, those who had results for polymerase chain reaction and serology, as well as those who came from a gold mining area in western French Guiana, were selected for the current analysis

Among the 801 participants in the cross-sectional surveys, 755 (94.3%) were included in this analysis (403 for ORPAL 1 and 352 for ORPAL 2), according to the selection criteria detailed in Fig. 2.

The median age of the participants was 38 years (min 18, max 70), and the sex ratio was 2.5 men per woman. The majority (94.8%) of the participants were born in Brazil, primarily from the Maranhão State (52.7%) or Pará and Tocantins states (23.6%).

About a half of the participants (54.3%) self-identified as gold miners strictly speaking, while the remainder were engaged in support activities, including individuals responsible for transporting personnel or shipping goods, mobile vendors, owners of small shops and restaurants located within the mining areas, and cooks. The median duration of involvement in gold mining was 10 years, and the median duration of activity at the most recent gold mining site was six months.

Further characteristics are delineated in Table 1.

**Table 1:** Description of the main socio-demographic and occupational characteristics of the participants in the cross-sectional surveys carried out in 2015 and 2019 at the French-Surinamese border, including people who have recently returned from a gold mining site in western French Guiana

		Overall (755)		2015—ORPAL 1 (403)		2019—ORPAL 2 (352)	
		Number (%) or Median (minimum–maximum)		Number (%) or Median (minimum–maximum)		Number (%) or Median (minimum–maximum)	
Age	18–29 years	157	(20.8%)	88	(21.8%)	69	(19.6%)
	30–44 years	361	(47.8%)	204	(50.6%)	157	(44.6%)
	Over 45	237	(31.4%)	111	(27.5%)	126	(35.8%)
Gender	Woman	215	(28.5%)	120	(29.8%)	95	(27.0%)
	Men	540	(71.5%)	283	(70.2%)	257	(73.0%)
Place of birth	Maranhão state (Brazil)	398	(52.7%)	210	(52.1%)	188	(53.4%)
	Pará or Tocantins states (Brazil)	178	(23.6%)	94	(23.3%)	84	(23.9%)
	Amapá state (Brazil)	45	(6.0%)	23	(5.7%)	22	(6.2%)
	Other state in the Northeast of Brazil	42	(5.6%)	22	(5.5%)	20	(5.7%)
	Other state in the North of Brazil	18	(2.4%)	13	(3.2%)	5	(1.4%)
	Other state of Brazil	35	(4.6%)	16	(4.0%)	19	(5.4%)
	Outside Brazil	39	(5.2%)	25	(6.2%)	14	(4.0%)
Level of education	*No formal education*	45	(6.0%)	26	(6.5%)	19	(5.4%)
	Primary	267	(35.4%)	164	(40.7%)	103	(29.3%)
	Secondary	425	(56.3%)	204	(50.6%)	221	(62.8%)
	University level education	15	(2.0%)	7	(1.7%)	8	(2.3%)
	Don’t know	3	(0.4%)	2	(0.5%)	0	(0.0%)
Time already spent working in gold mining (years)	5 years or less	223	(29.5%)	115	(28.5%)	108	(30.7%)
	6–10 years	200	(26.5%)	122	(30.3%)	78	(22.2%)
	11–15 years	132	(17.5%)	81	(20.1%)	51	(14.5%)
	More than 15 years	200	(26.5%)	85	(21.1%)	115	(32.7%)
Distance from last gold mining site to inclusion site (actual travel time)	Less than 2 h	68	(9.0%)	40	(9.9%)	28	(8.0%)
	Between 2 h and half a day	231	(30.6%)	120	(29.8%)	111	(31.5%)
	One day	191	(25.3%)	105	(26.1%)	86	(24.4%)
	More than one day	227	(30.1%)	137	(34%)	90	(25.6%)
	Don't know	32	(4.2%)	1	(0.2%)	31	(8.8%)
	ND	6	(0.8%)	0	(0.0%)	6	(1.7%)
Time spent on last gold mining site (in years)	ND Overall = 8; Orpal 1 = 3; Orpal 2 = 5	0.5	(0–16)	0.54	(0.02–15)	0.42	(0–16)
Main occupation category	Mining	410	(54.3%)	196	(48.6%)	214	(60.8%)
	Trade	145	(19.2%)	90	(22.3%)	55	(15.6%)
	Cooking and sex work	113	(15%)	62	(15.4%)	51	(14.5%)
	Logistics	65	(8.6%)	45	(11.2%)	20	(5.7%)
	Other	22	(2.9%)	10	(2.5%)	12	(3.4%)
Mobile profession	Mobile	177	(23.4%)	112	(27.8%)	65	(18.5%)
	Non-mobile	576	(76.3%)	291	(72.2%)	285	(81.0%)
	ND	2	(0.3%)	0	(0.0%)	2	(0.6%)
Reported lifelong number of malaria symptoms episodes	1 episode	81	(10.7%)	34	(8.4%)	47	(13.4%)
	2–3 episodes	90	(11.9%)	54	(13.4%)	36	(10.2%)
	4–7 episodes	66	(8.7%)	33	(8.2%)	33	(9.4%)
	More than 7 episodes	384	(50.9%)	238	(59.1%)	146	(41.5%)
	History of malaria but number unknown	3	(0.4%)	0	(0.0%)	3	(0.9%)
	None	131	(17.4%)	44	(10.9%)	87	(24.7%)
Reported malaria symptoms episode within the last 9 months	No	610	(80.8%)	288	(71.5%)	322	(91.5%)
	Yes	145	(19.2%)	115	(28.5%)	30	(8.5%)

The characteristics are presented in frequency (proportions) and median value (minimum, maximum)

### Malaria transmission

**Fig. 3:**
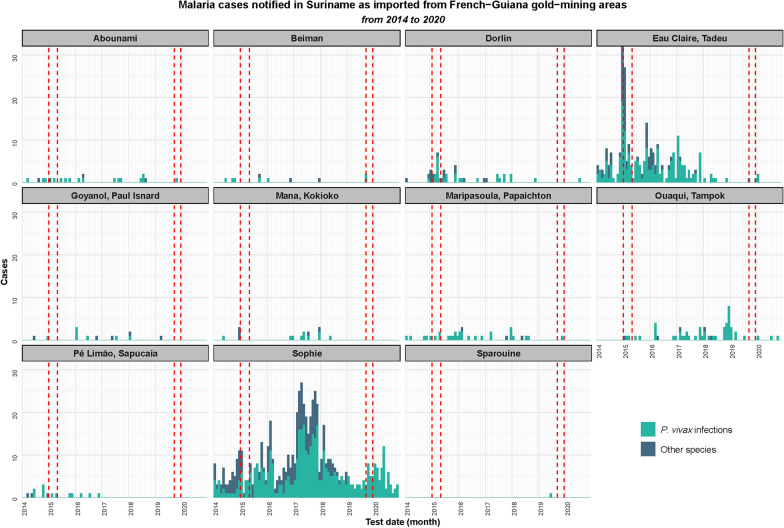
Epidemiological curves representing malaria cases notified in Suriname as imported from western French Guiana gold mining areas from 2014 to 2020, according to identified *Plasmodium* species (*P. vivax* infection mixed or alone in green, versus other species in blue). Case-per-month curves are represented for each gold mining area, and the periods during which the cross-sectional surveys were performed are presented within the red strokes lines

The importation of malaria cases from French Guiana gold mining areas to Suriname decreased between the beginning and the end of the analysed period. The number of malaria cases imported from French Guiana gold mining areas, as described by Suriname surveillance data, increased from 236 in 2014 to a peak of 487 in 2017 (related to an outbreak primarily concentrated in the Sophie mining region, see Fig. 3), before decreasing to 74 in 2020. The proportion of *P. vivax* among imported cases increased from 52.4% in 2014 to 100% in 2020. The annual figures are presented in the Supplementary Materials.

The proportion of survey participants testing positive for malaria by PCR decreased from 23.1% to 5.4% for all species combined (Table 2). With regard to *P. vivax* positive samples (alone or mixed infection), this value fell from 11.4% to 4.0%. Concurrently, the seroprevalence of *P. vivax* (SEM: 63–90) decreased from 44.7% to 28.4%. Amongst individuals who were positive by PCR, the proportion of *P. vivax* infections rose from 49.5% to 73.6% during the study period.

**Table 2:** PCR (polymerase chain reaction) and serology results for ORPAL 1 and ORPAL 2 blood samples

		2015	2019
PCR positivity	*Plasmodium falciparum*	10.9% (44)	0.9% (3)
	*P. vivax*	8.7% (35)	4.0% (14)
	*P. malariae*	0.7% (3)	0.6% (2)
	*P. falciparum* + *P. vivax*	2.5% (10)	0.0% (0)
	*P. vivax* + *P. malariae*	0.2% (1)	0.0% (0)
*P. vivax* seropositivity (SEM: 63–90)		44.7% (180)	28.4% (100)
*P. vivax* seropositivity (SEM: 79–79)		67.5% (272)	53.7% (189)

Data are presented in proportion (frequency)

As demonstrated by the epidemic curves in Fig. 3 and the proportions of positive samples in Table 3, the number of sites with evidence of transmission decreased over the study period. The cross-sectional survey data revealed an increase in *P. vivax* positivity exclusively in the Abounami and Sophie mining regions. However, none of these changes were found to be statistically significant.

**Table 3:** Proportion of participants with *Plasmodium vivax* positive PCR (polymerase chain reaction) per gold mining region and per survey. Data are presented in proportion and frequency (numerator/denominator)

Gold mining region	Positive PCR for *P. vivax*	
	2015	2019
Abounami	3.8% (1/26)	6.7% (4/60)
Beiman	0.0% (0/33)	0.0% (0/4)
Dorlin	9.5% (4/42)	0.0% (0/22)
Eau Claire, Tadeu	10.5% (13/124)	2.7% (2/74)
Goyanol, Paul Isnard	14.3% (2/14)	0.0% (0/17)
Mana, Kokioko	11.5% (6/52)	5.6% (1/18)
Maripa Soula, Papachton	20.0% (4/20)	3.6% (1/28)
Ouaqui, Tampok	11.1% (2/18)	1.3% (1/79)
Pe de limão, Sapucaia	6.7% (1/15)	0.0% (0/1)
Sophie	22.8% (13/57)	31.2% (5/16)
Sparouine	0.0% (0/2)	0.0% (0/33)

### Seropositivity and associated factors

*P. vivax* SEM 79–79 positivity was observed in 60.3% of survey participants (67.5% in 2015 and 53.7% in 2019).

In the multivariable regression analysis, the following characteristics were positively associated with seropositivity: increasing age, more than 5 years worked in gold mining, male gender, reporting an episode of malaria symptoms in the last nine months [odds ratio (*OR*) = 10.73, 95% confidence interval (*CI*): 5.87–19.6] or reporting older episodes of malaria symptoms [(*OR* = 2.22, 95% *CI*: 1.27–3.89) for one to three episodes, (*OR* = 5.31, 95% *CI*: 3.13–9.01) for more than three episodes]. The various occupational categories exhibited a significantly heterogeneous risk of seropositivity (see Table 4).

**Table 4:** Bivariable and multivariable analysis based on seropositivity (SEM 79–79) for *Plasmodium vivax*

		SEM 79–79 Negative			SEM 79–79 Positive			Bivariable analysis	Multivariable analysis	
		Frequency or mean (*SD*)	(%) or Median (min–max)	NAs	Frequency or mean (*SD*)	(%) or Median (min–max)	NAs	*P*-value	a*OR* (95% *CI*)	*P*-value
Survey year	2015	163/352	(46.3%)	0	189/352	(53.7%)	0	< 0.001		
	2019	131/403	(32.5%)		272/403	(67.5%)				
Age (years)		35.94 (10.24)	35 (18–64)	0	40.75 (9.96)	41 (18–70)	0	< 0.001	1.03 (1.01–1.05)	0.003
Sex	Woman	103/215	(47.9%)	0	112/215	(52.1%)	0	0.002	1	0.02
	Men	191/540	(35.4%)		349/540	(64.6%)			2.18 (1.10–4.31)	
Level of education	No school attendance or up to primary level	90/314	(28.7%)	0	224/314	(71.3%)	1	< 0.001		
	Secondary or university level education	204/440	(46.4%)		236/440	(53.6%)				
Reported clinical malaria history	Never	103/131	(78.6%)	0	28/131	(21.4%)	0	< 0.001	1	< 0.001
	1–3 old malaria symptoms episodes	74/144	(51.4%)		70/144	(48.6%)			2.22 (1.27–3.89)	
	More than 3 old malaria symptoms episodes	87/335	(26.0%)		248/335	(74.0%)			5.31 (3.13–9.01)	
	At least one malaria symptoms episode in the last 9 months	30/145	(20.7%)		115/145	(79.3%)			10.73 (5.87–19.6)	
Time spent in gold mining (years)	5 years or less	141/223	(63.2%)	0	82/223	(36.8%)	0	< 0.001	1	0.003
	6–10 years	69/200	(34.5%)		131/200	(65.5%)			2.12 (1.35–3.33)	
	11–15 years	32/132	(24.2%)		100/132	(75.8%)			2.86 (1.65–4.94)	
	More than 15 years	52/200	(26.0%)		148/200	(74.0%)			2.06 (1.18–3.58)	
Main occupation	Trade	47/145	(32.4%)	0	98/145	(67.6%)	0	0.005	1	0.04
	Mining	164/410	(40.0%)		246/410	(60.0%)			0.47 (0.25–0.88)	
	Cooking and/or sex work	57/113	(50.4%)		56/113	(49.6%)			0.72 (0.38–1.34)	
	Logistics	16/65	(24.6%)		49/65	(75.4%)			0.96 (0.43–2.17)	
	Other	10/22	(45.5%)		12/22	(54.5%)			0.71 (0.22–2.24)	
Number of mines were he/she worked in last 3 years		3.49 (3.53)	2 (1–31)	0	3.66 (3.39)	3 (1–30)	0	0.02		
Number of travels outside the mining area in the last 12 months	From 0 to 2	117/254	(46.1%)	3	137/254	(53.9%)	3	0.005		
	From 2 to 4	55/176	(31.2%)		121/176	(68.8%)				
	From 4 to 8	48/150	(32%)		102/150	(68.0%)				
	Only 8 more to go	71/169	(42%)		98/169	(58.0%)				
Mosquito net declared use (last night)	No	198/540	(36.7%)	0	342/540	(63.3%)	0	0.047		
	Yes	96/215	(44.7%)		119/215	(55.3%)				

Variables are described by frequency (proportion) for categorical variables, or by mean (standard deviation) and median (minimum**–**maximum) values for quantitative variables**.** Results of the analyses are expressed in *P*-values, adjusted odds ratios (a*OR*) and their 95% confidence intervals (*CI*)

**Fig. 4:**
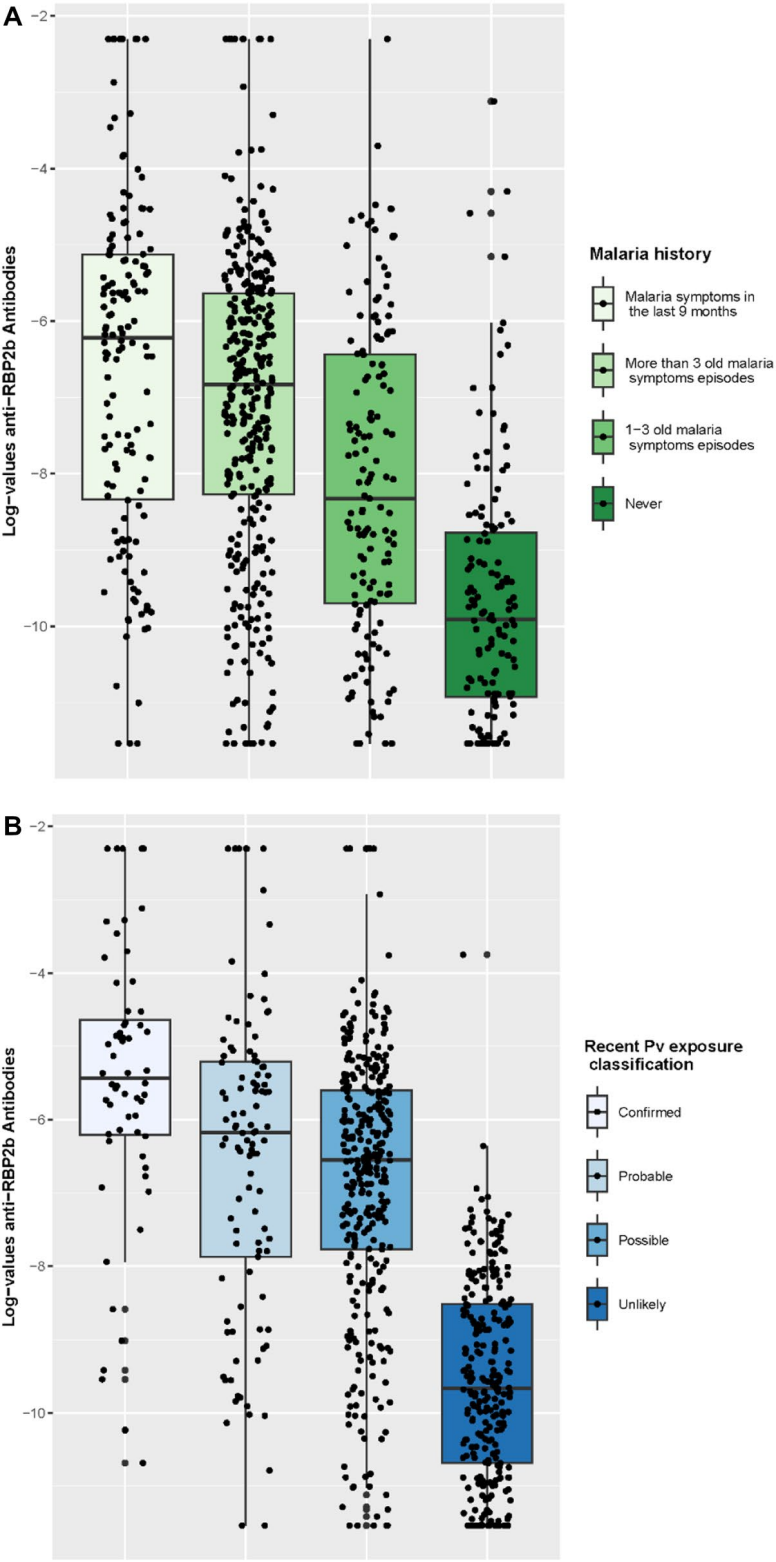
Distribution of anti-RBP2b.F2 log-value among participants in cross-sectional surveys categorized according to the reported malaria history (**A**) and according to the classification for estimating likelihood of recent *Plasmodium vivax* (Pv) infection (**B**)

The distributions of anti-RBP2b.F2 antibody levels (log value) in the study population, according to the reported history of malaria symptoms and according to the proposed estimated likelihood of recent *P. vivax* infection, are illustrated in Fig. 4.

### Assessment of likelihood of *P. vivax* recent infection

According to the composite indicator developed to estimate the probability of recent exposure to *P. vivax*, 8.0% of the surveys participants were classified as having a confirmed exposure because they were carriers of blood-stage forms (symptomatic or asymptomatic) (11.4% in 2015 and 4% in 2019); 13.9% had a probable recent exposure (19.9% in 2015 and 7.1% in 2019); 43.6% had a possible exposure (42.5% in 2015 and 44.9% in 2019); finally, 34.6% had an unlikely exposure (26.3% in 2015 and 44.0% in 2019).

Table 5 provides a more detailed description of this indicator and compares it with the biological or epidemiological tools that may be used to select individuals to be treated in interventions aimed at reducing the human reservoir of *P. vivax* malaria. While PCR is capable of detecting a significant number of blood-stage parasite carriers (81.6% of whom are asymptomatic), it is unable to detect individuals with (probable or possible) recent exposure, and potential hypnozoite carriers. For comparison, 57/60 (95.0%) of survey participants with confirmed exposure, 92/105 (87.6%) with probable exposure and 312/329 (94.8%) with possible exposure were positive by serology (79% Se –79% Sp). According to the epidemiological criteria currently employed for TDA against *P. vivax* in the CUREMA project, 57/60 (95%) of participants with confirmed exposure, 105/105 (100%) with probable exposure and 303/329 (92.1%) with possible exposure, but also 159/261 (60.9%) with unlikely exposure, would be eligible for such a treatment.

**Table 5:** Composite indicator for estimating the likelihood of recent *Plasmodium vivax* infection, and its comparison with biological or epidemiological criteria that can be used to select people to be treated in interventions aimed at reducing the human reservoir of malaria

		Participants per catergory			Participants selected for treatment per category according to:				
		2015	2019	Overall	*P. vivax* PCR	SEM 79–79	SEM 63–90	Reported malaria history in the last 9 months	CUREMA TDA eligibility criteria
Confirmed	Symptomatic and PCR positive	8 (2.0%)	3 (0.9%)	**11** **(1.5%)**	11/11 (100%)	10/11 (90.9%)	6/11 (54.5%)	7/11 (63.6%)	10/11 (90.9%)
	Asymptomatic and PCR positive	38 (9.4%)	11 (3.1%)	**49** **(6.5%)**	49/49 (100%)	47/49 (95.9%)	41/49 (83.7%)	16/49 (32.6%)	47/49 (95.9%)
Probable	PCR negative, reporting malaria-like symptoms with positive testing in the last 9 months	27 (6.7%)	19 (5.4%)	**46** **(6.1%)**	0/46 (0.0%)	33/46 (71.7%)	21/46 (45.6%)	46/46 (100%)	46/46 (100%)
	PCR negative, reporting malaria-like symptoms without a biological diagnosis in the last 9 months but with positive SEM (79–79)	53 (13.2%)	6 (1.7%)	**59** **(7.8%)**	0/59 (0.0%)	59/59 (100%)	45/59 (76.3%)	59/59 (100%)	59/59 (100%)
Possible	PCR negative, not reporting any malaria-like symptoms in the last 9 months but SEM (79–79) positive	155 (38.5%)	157 (44.6%)	**312** **(41.3%)**	0/312 (0.0%)	312/312 (100%)	167/312 (53.5%)	0/312 (0.0%)	286/312 (91.7%)
	PCR negative, reporting malaria-like symptoms in the last 9 months but SEM (79–79) negative	16 (4.0%)	1 (0.3%)	**17** **(2.3%)**	0/17 (0.0%)	0/17 (0.0%)	0/17 (0.0%)	17/17 (100%)	17/17 (100%)
Unlikely	PCR negative, SEM (79–79) negative and not reporting any history of malaria symptoms in the last 9 months	106 (26.3%)	155 (44%)	**261 (34.6%)**	0/261 (0.0%)	0/261 (0.0%)	0/261 (0.0%)	0/261 (0.0%)	159/261 (60.9%)

Bold indicates make more visible the column with the frequencies for overall study population, which are the denominators for the proportions calculated in the second part of the table

The Supplementary Materials present a graphical representation of the proportions that each of these tools allows to be obtained among the individuals considered to be treated (confirmed and probable exposure), those considered not to be treated (unlikely exposure) and those for whom there is still some doubt (possible exposure).

## Discussion

This article examines trends in malaria circulation in recent years in a hard-to-reach population, one of the last relevant pockets of transmission in a region approaching elimination.

### A reduction of malaria transmission over the study period

The data analysed describe a significant reduction in malaria transmission, visible in both cross-sectional studies (PCR and serology results, number of recent malaria episodes reported by participants) and surveillance data. This reduction is linked to the considerable efforts made to strengthen malaria control in the study areas. Specific interventions targeting the population involved in AGSM took place during the period analysed, such as the activity in Suriname of a network of community health workers (malaria service deliverers [MSDs]) created in the mining sector in 2006 as part of the project “Looking for gold, finding malaria” [28, 29], and the international Malakit research project between 2018 and March 2020 [25]. The latter provided self-testing and self-treatment kits, together with health education activities, to gold miners working in mining sites located in French Guiana. This intervention had a significant impact on the management of malaria symptoms in the study population, and on malaria transmission more generally. The content of the kit in terms of treatment (artemether-lumefantrine and single low-dose primaquine) makes it particularly suitable for controlling the cycle of *P. falciparum* [24, 30].

During the period analyzed an increase in the proportion of cases due to *P. vivax* and a decrease in the number of areas with active transmission (higher spatial heterogeneity) were observed. This trend, as well as the appearance of epidemiological instability associated with outbreaks, is expected when malaria transmission decreases [31–33].

A comparison of survey data with surveillance data reveals some discrepancies in sectors with active transmission. There are several possible explanations for these differences. First, the cross-sectional surveys were not designed to describe a geographical stratification of transmission, so the numbers are insufficient to obtain accurate estimates. In addition, the cross-sectional survey data are based on the systematic analysis of a large number of asymptomatic participants using molecular biology methods, much more sensitive than those (RDTs and microscopy) used to confirm cases reported by the Surinamese surveillance system. It is also possible that some of the cases were captured by the French surveillance system; the lack of information in the French surveillance system on gold mining activities and on the geographical details of the gold mining sector meant that we were unable to use these data in this analysis. Overall, these results demonstrate the complementary nature of these two approaches to describing transmission, and the value of systematic surveys and molecular biology in regions of low to moderate transmission.

### Value of *P. vivax* serology

The use of serology complements the data derived from the detection of blood-stage parasites (RDT, microscopy and PCR). In our study, SEM 63–90 data provide a general description of the intensity of the recent exposure of the population to *P. vivax*: it has decreased but the prevalence of exposed gold-miners was still considerable.

However, in the perspective of using the SEM to select individuals for treatment in the context of malaria elimination interventions, questions may arise in this epidemiological context. In our study population, the circulation of *P. vivax* is still significant, and has been very high until recently. For some of the gold miners, exposure to malaria may have occurred in their previous places of residence in Brazil, even before joining the gold mining business. For older participants, and for those with a longer history of gold mining in the Guiana Shield and the Amazon region, the history of exposure to malaria may therefore be intense. This is reflected in the study data: age, time spent in gold mining and past history of malaria have a significant influence on the SEM 79–79 results. This can also be illustrated by the distribution of anti-RBP2b.F2 antibodies in our study population according to the reported recent and longstanding history of malaria.

The analysis of seropositivity was not explored in geographical detail because the mobility data available referred only to the last gold mining area frequented: in a context of high mobility (median duration of presence 6 months) these spatial data therefore did not have sufficient chronological depth.

### Criteria for identifying asymptomatic *P. vivax* carriers

Our study suggests a classification of participants according to the likelihood of recent *P. vivax* infection. This is seen as a proxy for the risk of being a carrier of the parasite (either asymptomatic blood stage or hypnozoite carrier) in order to identify people who might benefit from antimalarial drug administration in population-based interventions. This classification is undoubtedly questionable, but it attempts to take into account of all the data that may be available in cross-sectional surveys, which by design suffer from a lack of biological and clinical follow-up data. The proposed classification should therefore be validated by longitudinal data from comparable populations.

Nevertheless, there are a number of interesting points to be made about this classification. First, it illustrates how individuals identified through passive detection (symptomatic individuals) represent a very small tip of the iceberg of potential hypnozoite carriers. Even an active case detection strategy based on systematic screening using sophisticated molecular biology methods (which are often not widely available in the field) would only be able to capture a fraction of these cases. In this context, the cost-effectiveness of using these strategies to eliminate the human parasite reservoir is questionable.

In our sample, the use of SEM 79–79 would allow the selection of almost all PCR-positive individuals, and the majority of individuals reporting a recent history of malaria. The choice of these thresholds therefore appears to represent a significant gain in sensitivity when aiming to eliminate malaria, but at the cost of a larger proportion of people being wrongly treated compared with SEM 63–90.

Furthermore, in our sample it can be observed that the introduction of a simple epidemiological criterion, such as that used in the CUREMA project’s intervention TDA (collection of information on the recent history of malaria symptoms, associated with recent exposure to active transmission areas), makes it possible to identify almost all the participants with a positive PCR and a significant proportion of those with a positive SEM 79–79. On the other hand, this criterion is weighed down by a relatively high proportion of individuals who may have been wrongly treated. The TDA selection criterion chosen for the CUREMA project attempts to combine satisfactory sensitivity with ease of use in the field when interviewing participants. In fact, it uses broad geographical definitions (linked to the difficulty in the field of asking individuals about their complete itineraries in contexts of toponymic ambiguity) and, for the sake of simplicity, extends the search for exposure to places of active transmission in the previous year. It will be further evaluated in the cross-sectional post-intervention survey of the study.

The lack of longitudinal data means that the categories constructed to estimate the probability of *P. vivax* infection are compared with the criteria used to define them: this approach therefore has limitations that must be taken into account. Despite the limitations of this analysis, it seems to indicate that the use of SEM results with sufficient sensitivity would be an ideal compromise for selecting patients for treatment in these settings. Nevertheless, in field settings where it is not possible to use laboratory results and while serological RDTs are not available, taking into account clinical and epidemiological history is a cheap and simple selection method that is able to capture the majority of the highly probable carriers. Even if this selection is associated with a significant proportion of people still being treated unnecessarily, the expected risk and cost–benefit would be better than administering drugs to the whole high-risk population.

Furthermore, although modelling studies have been carried out, it remains to be investigated in real life what proportion of these asymptomatic carriers will go on to contribute to disease transmission and what the real benefit of systematic treatment would be [11, 16].

The significant association found in these data between the reported history of malaria symptoms and its biological markers (PCR or serology) seems to support the observation from qualitative studies that symptomatic forms of malaria seem to be relatively well recognized in the community studied, despite the many potential differential diagnoses. This seems to be related to the individual and collective experience of the disease and to the cultural background of this Amazonian population, which has historically been subject to the burden of malaria [34].

## Conclusions

As in many other contexts, the reduction in malaria transmission in the gold mining sector in French Guiana is accompanied by the difficulty of targeting the last pockets of *P. vivax* transmission in an effective and balanced way. Identifying and treating the silent human reservoir of this parasite is a relevant way to achieve this goal, and the lessons learned may be useful in many contexts facing similar challenges, in South America and elsewhere in the world.

## Supplementary Information

Additional file 1

## Data Availability

The anonymized datasets generated and/or analysed during the current study are not publicly available due given the particularly sensitive nature of these data concerning a population carrying out an activity punishable by law, but are available from the corresponding author on reasonable request.

## Abbreviations

ASGM: Artisanal and small-scale gold mining
*CI*: Confidence interval
CUREMA: Radical CURE for MAlaria among highly mobile and hard-to-reach populations in the Guiana Shield
FG: French Guiana
IPG: Institut Pasteur de la Guyane
MDA: Mass drug administration
MSD: Malaria service deliverer
*OR*: Odds ratio
ORPAL: Orpaillage et paludisme—gold mining and malaria
PCR: Polymerase chain reaction
RBP2b: Reticulocyte binding protein 2b
RDT: Rapid diagnostic test
SEM: Serological exposure markers
SeroTaT: Serological test and treatment
TDA: Targeted drug administration
WHO: World Health Organization
