# Supplementary material for: Assessment of Plasmodium vivax transmission and asymptomatic carriage risk among artisanal gold miners in western French Guiana, 2014–2020
Source: Infect Dis Poverty. 2025 May 26;14:40. doi: 10.1186/s40249-025-01306-8 (PMC12105214; doi:10.1186/s40249-025-01306-8)

**Supplementary Materials**

|  | Malaria cases | P. vivax positive cases |
| --- | --- | --- |
| 2014 | 201 | 103 (51.2%) |
| 2015 | 251 | 175 (69.7%) |
| 2016 | 212 | 130 (61.3%) |
| 2017 | 478 | 347 (72.6%) |
| 2018 | 158 | 122 (77.2%) |
| 2019 | 68 | 67 (98.5%) |
| 2020 | 73 | 73 (100%) |

Table 1: Number of malaria cases notified as imported to Suriname from gold-mining areas in western French Guiana, between 2014 and 2020

Figure 1: Participants that would have been selected for treatment (in grey) according to different biological and epidemiological criteria, in each one of the three following categories: : (A) people with a confirmed or probable recent infection should be treated; (C) people with an unlikely recent infection should not be treated; (B) a doubt persist about the relevance of offering treatment to people with a possible recent infection.


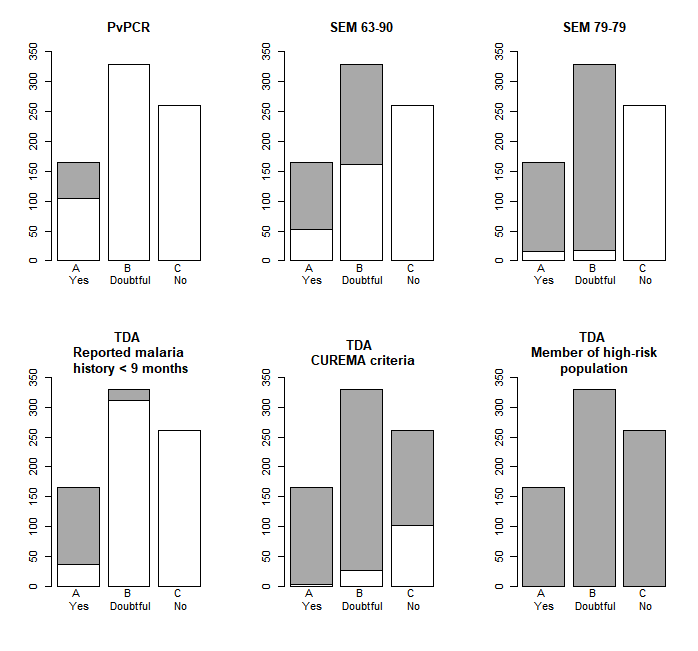

Supplement: Supplementary file 1 — Additional file 1 [file 40249_2025_1306_MOESM1_ESM.docx]
